# A novel registration-based methodology for prediction of trabecular bone fabric from clinical QCT: A comprehensive analysis

**DOI:** 10.1371/journal.pone.0187874

**Published:** 2017-11-27

**Authors:** Vimal Chandran, Mauricio Reyes, Philippe Zysset

**Affiliations:** Institute of Surgical Technology and Biomechanics, University of Bern, Bern, Switzerland; Rensselaer Polytechnic Institute, UNITED STATES

## Abstract

Osteoporosis leads to hip fractures in aging populations and is diagnosed by modern medical imaging techniques such as quantitative computed tomography (QCT). Hip fracture sites involve trabecular bone, whose strength is determined by volume fraction and orientation, known as fabric. However, bone fabric cannot be reliably assessed in clinical QCT images of proximal femur. Accordingly, we propose a novel registration-based estimation of bone fabric designed to preserve tensor properties of bone fabric and to map bone fabric by a global and local decomposition of the gradient of a non-rigid image registration transformation. Furthermore, no comprehensive analysis on the critical components of this methodology has been previously conducted. Hence, the aim of this work was to identify the best registration-based strategy to assign bone fabric to the QCT image of a patient’s proximal femur. The normalized correlation coefficient and curvature-based regularization were used for image-based registration and the Frobenius norm of the stretch tensor of the local gradient was selected to quantify the distance among the proximal femora in the population. Based on this distance, closest, farthest and mean femora with a distinction of sex were chosen as alternative atlases to evaluate their influence on bone fabric prediction. Second, we analyzed different tensor mapping schemes for bone fabric prediction: identity, rotation-only, rotation and stretch tensor. Third, we investigated the use of a population average fabric atlas. A leave one out (LOO) evaluation study was performed with a dual QCT and HR-pQCT database of 36 pairs of human femora. The quality of the fabric prediction was assessed with three metrics, the tensor norm (TN) error, the degree of anisotropy (DA) error and the angular deviation of the principal tensor direction (PTD). The closest femur atlas (CTP) with a full rotation (CR) for fabric mapping delivered the best results with a TN error of 7.3 ± 0.9%, a DA error of 6.6 ± 1.3% and a PTD error of 25 ± 2°. The closest to the population mean femur atlas (MTP) using the same mapping scheme yielded only slightly higher errors than CTP for substantially less computing efforts. The population average fabric atlas yielded substantially higher errors than the MTP with the CR mapping scheme. Accounting for sex did not bring any significant improvements. The identified fabric mapping methodology will be exploited in patient-specific QCT-based finite element analysis of the proximal femur to improve the prediction of hip fracture risk.

## Introduction

Osteoporotic hip fractures represent a major clinical and public health problem in aging populations. Identifying individuals at higher fracture risk would enable targeted osteoporosis management and improve fracture prevention. Areal bone mineral density (aBMD) measured by dual-energy x-ray absorptiometry (DXA) is routinely used as a surrogate of bone strength for osteoporosis diagnosis and fracture risk assessment. Modern techniques such as finite element (FE) analysis allow for a more accurate estimation of bone strength using the local distribution of BMD provided by QCT, but do not account for the anisotropy of trabecular bone architecture called fabric. Recent validation studies have demonstrated that the inclusion of bone fabric (anisotropy) in FEA models is important and delivers an improved prediction of bone strength [1–5]. However, measuring bone fabric requires high resolution peripheral QCT (HRpQCT) images and presently, this resolution is not available clinically for the proximal femur.

Consequently, computational approaches to accurately predict bone fabric directly from clinical QCT images are receiving increasing interest. In this regard, machine learning approaches have been recently used to predict bone fabric, where the statistical relationship between clinical QCT imaging information, and its corresponding high-resolution peripheral QCT (HRpQCT) was modelled, and then used to perform inference of bone fabric on (unseen) clinical QCT images. Particularly, discriminative models that infer bone fabric from computed features (i.e predictor variables) have been proposed. In [6], nodal displacements of a template mesh registered to a patient-specific mesh are used as features for a non-linear kernel partial least square (PLS) regression approach. In [7], morphology- and texture-based features are used as features as part of a decision forest regression approach. While these statistical approaches have showed promising results, they involve manual annotations of landmarks for initial alignment, and their accuracy depends on the selected training data.

Another family of approaches for bone fabric estimation is based on image or mesh registration. In [3], the authors rely on a database of HRpQCT-based derived FE models of femurs including bone density and fabric information. From the database, the most similar femur to the target femur is selected by means of mesh-morphing and a bone-mineral-density similarity metric computed across the database. Finally, the pre-computed fabric information of the selected femur is mapped to the patient’s femur by rigidly correcting the local orientation of the fabric information. It is noted, however, that this study did not perform a direct study on clinical CT images. Recently, in [2, 8], the authors investigated intensity based registration methods to derive fabric information. In their approach, rather than pre-computing fabric information and then mapping the closest femur in the database to the patient’s femur, bone fabric is inferred by registering a single QCT image to the patient’s image, and then it calculates fabric on the corresponding non-rigidly transformed HRpQCT image.

In these previous studies two issues are identified with respect to the chosen registration approach and the degrees of freedom of the transformation model used to map fabric information to the patient’s image. The study in [3] used a surface-based mesh morphing approach (using eight sparsely located landmarks), and a rotation-based local correction to map fabric information to the target image. It is first remarked that surface-based registration approaches have been reported to be less accurate than intensity-based registration approaches for establishing anatomical point correspondences [9]. Secondly, the study in [3] uses a local correction based on a rotation matrix, which is not proved to provide the best result in terms of fabric matching to a patient’s image. Similarly, the approaches in [2, 8] employ an image-intensity registration approach and the complete non-rigid transformation (i.e. no decomposition or local correction of the transformation) to derive fabric information. In this regard, as demonstrated in the present study, the degrees of freedom of the transformation model used to map fabric information to the patient’s image plays an important role on the accuracy of these methods.

Consequently, and differently from previous approaches, we propose a novel registration-based estimation of bone fabric directly from clinical QCT image. It is designed to preserve tensor properties of bone fabric and to map bone fabric by a global and local decomposition of the gradient of a non-rigid image registration transformation. Another issue investigated in this study comes from the fact that the role of utilising a database of femoral atlases, from which fabric information is mapped to a patient’s image, is not known and inconclusive from the state of the art. The conclusions presented in [2, 8] contradict with those of [3], on the fact that a single femur atlas might suffice to estimate femur fabric from a QCT patient image. These contradictory results might be amplified by the fact that these studies were tested on a very limited set of ten cases. In this study we therefore present a thorough leave-one-out analysis on the importance of atlas selection for bone fabric estimation on a dataset comprising 36 pairs of QCT and HRpQCT human femora. Using a deformation-based distance metric, we evaluate six different atlases that span different degrees of similarity to the target image, and are population-wide or sex-specific. In addition, beyond bone shape and image atlases, we evaluate and report on the ability of a single population-, and sex-specific atlases of bone fabric used within the proposed registration-based fabric estimation approach.

## Methods

In this section the proposed image registration based fabric prediction is presented, followed by the methodology and metrics proposed to select a femur atlas from a given population. The section continues then with the proposed methodology to decompose the image transformation and apply it to the precomputed fabric of the chosen atlas. The section finishes with the scheme and metrics used to evaluate the quality of the proposed fabric prediction approach.

**Fig 1:**
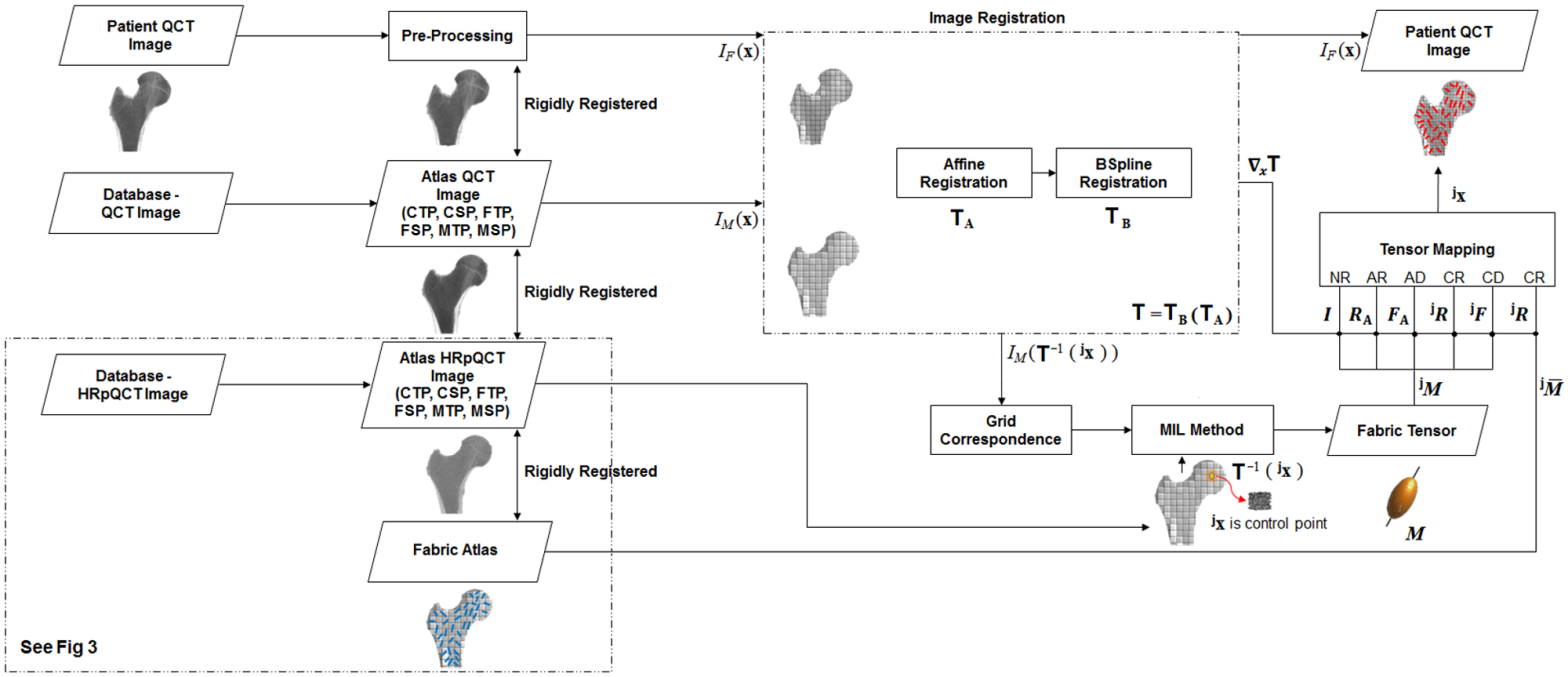
Overview of the image registration approach for predicting the bone fabric is presented. Different femur atlases (CTP,CSP,FTP,FSP,MTP,MSP) are used in the registration process and a detailed analysis is done for choosing the optimal femur atlas. Similarly, four different tensor mapping methods (CD,CR,AD,AR) are analyzed for mapping fabric tensor from the femur atlas to the patient’s femur QCT image. Alternatively, a fabric atlas (femur atlas model with mean fabric) is used for mapping bone fabric to patient’s femur QCT image and its impact is analyzed only for CR mapping method. Acronyms: CTP—Closest to patient femur in the Total Population, CSP—Closest to patient femur in the Sex-specific Population, FTP—Farthest to patient femur in the Total Population, FSP—Farthest to patient femur in the Sex-specific Population, MTP—Mean femur of the Total Population, MSP—Mean femur of the Sex-specific Population, NR—No rotation, AR—Affine Rotation, AD—Affine Deformation, CR—Complete Rotation, CD—Complete Deformation.

The complete overview of the registration approach in bone fabric prediction is presented in Fig 1 and is described in detail below.

### Image registration

Image registration is the process of aligning two images into a common coordinate system. Given a pair of images, a fixed image *I*_*F*_(***x***) and a moving image *I*_*M*_(***x***) are defined on their own spatial domain: $\Omega_{F}\subset R^{3}$ and $\Omega_{M}\subset R^{3}$, and here ***x*** = {*x*1, *x*2, *x*3} denotes the voxel location. Image registration is the task of finding a coordinate transform $T:R^{3}\to R^{3}$ that spatially aligns the two images such that a given similarity metric between *I*_*F*_(***x***) and *I*_*M*_(***T***(***x***)) is optimized [10]. Image registration can be formulated as an optimization problem:

$$\begin{array}{c} \hat{T}=argmin\;C\left(T;I_{F},I_{M}\right)=argmin\left(-C_{similarity}\left(T;I_{F},I_{M}\right)+\gamma C_{smooth}\left(T\right)\right). \end{array}$$ (1)

The cost function *C* defines the quality of alignment, which is separated into a similarity measure *C*_*similarity*_ and a regularization term *C*_*smooth*_. In this work, normalized correlation coefficient [10] is used as the similarity measure because of its ability to handle mono-modal image registration. Curvature regularization [11] is used as regularization term to cope with the ill-posedness of the non-rigid image registration. It acts on the deformation field computed on the B-Spline grid nodes. The parameter *γ* weighs regularity against similarity.

$$\begin{array}{c} C_{similarity}\left(T;I_{F},I_{M}\right)=\sum_{x\in \Omega }\frac{\left(I_{F}\left(x\right)-\bar{I_{F}}\right)\left(I_{M}\left(T\left(x\right)\right)-\bar{I_{M}}\right)}{\sqrt{{\left(I_{F}\left(x\right)-\bar{I_{F}}\right)}^{2}(I_{M}\left(T\left(x\right)\right)-\bar{I_{M}}{)}^{2}}}, \end{array}$$ (2)

$$\begin{array}{c} C_{smooth}\left(T\right)=∥\Delta_{x}T{∥}^{2} \end{array}$$ (3)

In the present study the image registration process is performed in two stages. First, an affine registration is performed to get a coarse global alignment of the entire anatomy. Second, a cubic B-Spline registration is used to yield a fine local alignment based on a grid of *J* control points. The transformations are combined by composition, as follows

$$\begin{array}{c} T\left(x\right)=T_{B}\left(T_{A}\left(x\right)\right)), \end{array}$$ (4)

where ***T***_***A***_ is the affine transform and ***T***_***B***_ is the B-spline transform.

Parameter tuning of the registration was performed heuristically and based on the quality of the registration. To this end, we computed the Dice coefficient between image masks, which are obtained via semi-manual segmentation of the HRpQCT images for which a simple image thresholding is feasible. The Dice coefficient is then calculated on image masks transformed (i.e. Eq (4)) and resampled to the QCT image space by nearest-neighbor interpolation. The accuracy of the image registration in terms of Dice coefficient [12, 13] was in average of 94±3%, and hence considered satisfactory for the rest of the analyses. Furthermore, changing the order of operation (i.e. HRpQCT masks were first resampled and then transformed for Dice coefficient calculation) did not significantly affect the accuracy of the transformation (p>0.05).

#### Selecting a femur atlas

A femur atlas is a QCT image chosen from the population. Since there are various possible candidate femur atlases available in the population, we propose a strategy for choosing it. In principle, a good atlas is such having minimal image deformation needed to warp the atlas image to each fixed image in the population. Inspired from the Frechet mean and related works proposed in computational anatomy [14–16], a distance metric *DM* is proposed herein to measure the extent of deformation. In the proposed image registration process (Fig 1), *I*_*F*_ corresponds to the patient’s femur QCT image, while *I*_*M*_ corresponds to the femur atlas QCT image.

The distance metric *DM* is calculated using the stretch tensor ^***j***^***G***_***V***_, which is computed on the grid of control points {*j* = 1,…*J*} spanned over the entire registered image. The computation of the stretch ^***j***^***G***_***V***_ involves the combined transformation of affine and B-spline transforms. We use ^***j***^***G***_***V***_ ≡ ***V*** for simplicity in the rest of the paper.

The deformation gradient ***F*** is computed, which is the gradient of the transformation or the Jacobian matrix of the mapping

$$\begin{array}{c} F=\nabla_{x}T. \end{array}$$ (5)

Performing ***VR*** decomposition

$$\begin{array}{c} F=VR, \end{array}$$ (6)

$$\begin{array}{c} V={\left(FF^{T}\right)}^{1/2}, \end{array}$$ (7)

The distance metric *DM* is defined as

$$\begin{array}{c} DM=\sum_{j=1}^{J}||\log({}^{j}V)||=\sum_{j=1}^{J}||\log({}^{j}V_{B}{}^{j}V_{A})||, \end{array}$$ (8)

where ***V***_***A***_ and ***V***_***B***_ denotes the principal stretch of the affine and B-spline transforms, respectively.

**Fig 2:**
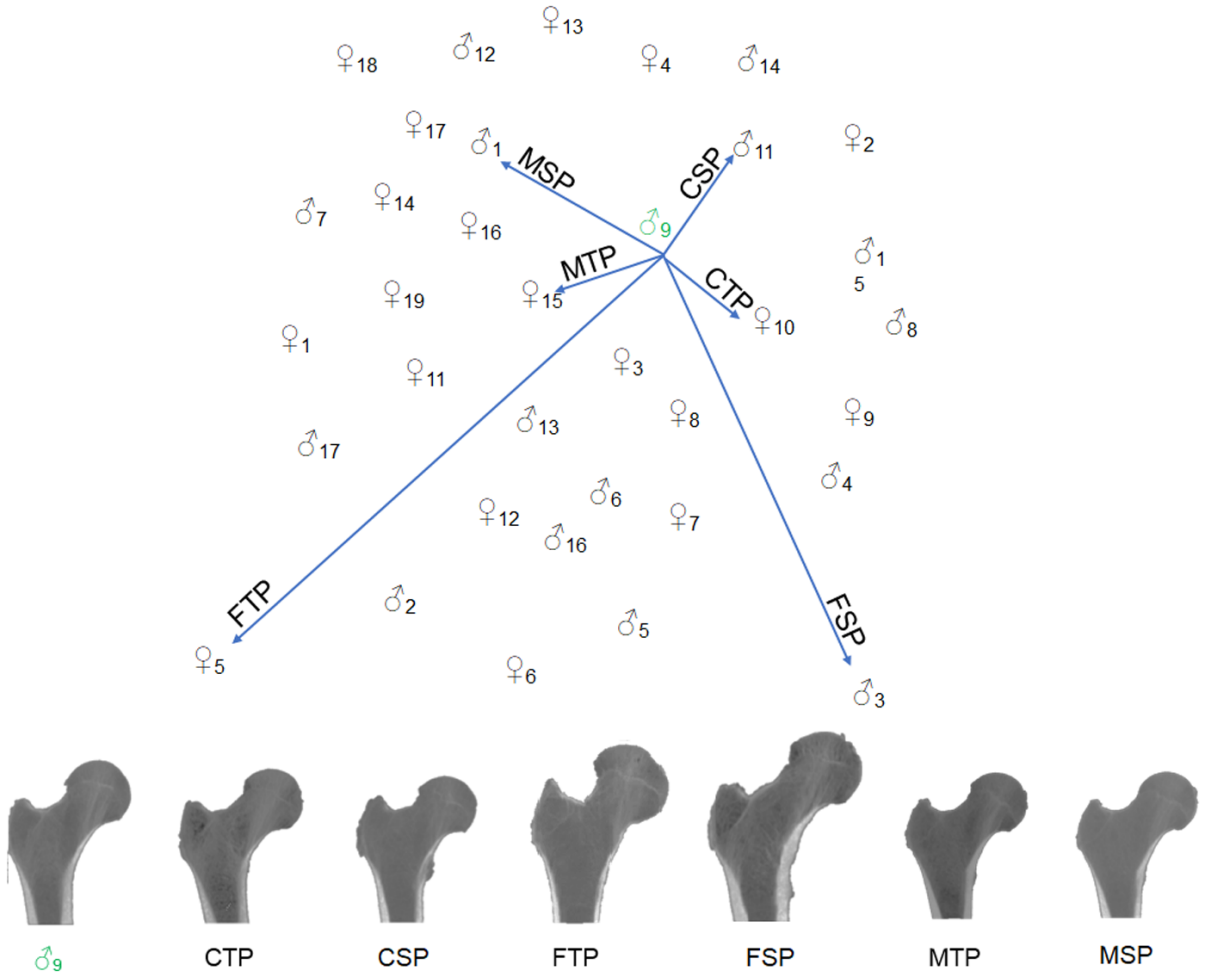
Femur atlas selection strategy. Top: Selection of different femur atlases from a population for registration with the patient’s femur. The selection was based on distance metric (DM). Bottom: Example—Coronal view of different selected femur atlases for a test case. Acronyms: CTP—Closest to patient femur in the Total Population, CSP—Closest to patient femur in the Sex-specific Population, FTP—Farthest to patient femur in the Total Population, FSP—Farthest to patient femur in the Sex-specific Population, MTP—Mean femur of the Total Population, MSP—Mean femur of the Sex-specific Population.

The distance metric *DM* is then used to select different atlases featuring different degrees of similarity to the target fixed image. Six different and representative femur atlases were chosen to evaluate the importance of selecting an appropriate femur atlas (see Fig 2). For concision, they are henceforth referred to as:

1. *Closest to the patient femur in the population (CTP,CSP)*: This atlas image corresponds to the femur image yielding the minimum distance metric (hence referred as closest to the patient’s femur). If *N* represents the total number of femurs in the population, then the **C**losest to the patient’s femur in the **T**otal **P**opulation, termed here *CTP*, is
  $$\begin{array}{c} CTP=min(\sum_{q=1}^{N}DM\left({}^{patient}I_{F},{}^{q}I_{M}\right)). \end{array}$$ (9)
  Similarly, if *N*_*sex*_ represents the total number of femurs in the sex-specific population, then the **C**losest to the patient’s femur in the **S**ex-specific **P**opulation, termed here *CSP*, is
  $$\begin{array}{c} CSP=min(\sum_{q=1}^{N_{sex}}DM\left({}^{patient}I_{F},{}^{q}I_{M}\right)). \end{array}$$ (10)
2. *Farthest to the patient femur in the population (FTP,FSP)*: The femur image yielding the maximum distance metric is considered to be the farthest to the patient’s femur. If *N* represents total number of femurs in the population, then the **F**arthest to the patient’s femur in the **T**otal **P**opulation, termed here *FTP*, is
  $$\begin{array}{c} FTP=max(\sum_{q=1}^{N}DM\left({}^{patient}I_{F},{}^{q}I_{M}\right)). \end{array}$$ (11)
  Similarly, if *N*_*sex*_ represents the total number of femurs in the sex-specific population, then the **F**arthest to the patient’s femur in the **S**ex-specific **P**opulation, termed here *FSP*, is
  $$\begin{array}{c} FSP=max(\sum_{q=1}^{N_{sex}}DM\left({}^{patient}I_{F},{}^{q}I_{M}\right)). \end{array}$$ (12)
  We note that inclusion of this femur as potential atlas is meant to provide a worst-case scenario, where the atlas and the patient’s femur are considerably geometrically different.
3. *Mean femur of the population (MTP,MSP)*: Generally, the mean femur of the population is a synthetic image produced through arithmetic computation [17]. However, such synthetic images are prone to present blurred intensity patterns of the femur fabric, stemming from the averaging process. Hence, we chose as mean femur atlas, the real femur image yielding the minimum accumulated distance metric across the population. If *N* represents the total number of femurs in the population, then the **M**ean femur of the **T**otal **P**opulation, termed here *MTP*, is
  $$\begin{array}{c} MTP=min(\sum_{p=1}^{N}\sum_{\begin{array}{c} q=1\\ p\neq q \end{array}}^{N}DM\left({}^{p}I_{F},{}^{q}I_{M}\right)). \end{array}$$ (13)
  Similarly, if *N*_*sex*_ represents the total number of femurs in the Sex-specific population, then **M**ean femur of the **S**ex-specific **P**opulation, termed here *MSP*, is
  $$MSP=min(\sum_{p=1}^{N_{sex}}\sum_{\begin{array}{l} q=1\\ p\neq q \end{array}}^{N_{sex}}DM{(}^{p}I_{F}{,}^{q}I_{M})).$$ (14)

### Bone fabric extraction

In this section we briefly describe the step of extracting and modeling fabric information. Bone fabric describes the preferential alignment and structural anisotropy of bone trabecular micro-architecture. It is computed using the MIL method [18], which measures the average distances of bone-marrow interfaces in multiple orientations on a segmented image. In summary, a Laplace Hamming filter is first applied to sharpen the HRpQCT image, which is then normalized, and segmented based on image thresholding [19]. On the segmented image, a cubic volume of interest (VOI) with a side length of 5.3mm is extracted, at each corresponding control point *I*_*M*_(***T***^−1^(^*j*^***x***)), from the trabecular region, and fabric tensor ^*j*^***M*** is computed using the MIL method.

The resulting spatial distribution can be described with a second-order fabric tensor $M\in R^{3X3}$ with eigenvalues *m*_*i*_ and normalized eigenvectors ***m***_***i***_.

$$\begin{array}{c} M=\sum_{i=1}^{3}m_{i}\left(m_{i}⊗m_{i}\right) \end{array}$$ (15)

where *m*_1_ ≤ *m*_2_ ≤ *m*_3_. The fabric tensor ***M*** is normalized by dividing it by its trace and multiplying it by a factor of 3 such that

$$\begin{array}{c} tr(M)=3. \end{array}$$ (16)

The shape of the fabric tensor can be visualized as an ellipsoid with magnitude of eigenvalues providing the indication of the extent to which the structure is preferentially aligned. An elongated ellipsoid represents an anisotropic structure (high degree of anisotropy) whereas a sphere represents an isotropic structure (absence of anisotropy).

#### Fabric tensor mapping

Computing the fabric tensor directly on the atlas image, which is transformed to the patient’s image via registration (e.g. as in [2, 8]) might result in loss of information as the registration process, involving local image deformations, tends to alter the bone fabric pattern. Contrarily, rather than computing fabric tensors on a transformed atlas image, we propose to map fabric information from the atlas to the patient image by transforming its tensorial representation instead. This is inspired by similar strategies followed in neuroimaging, for DTI image registration [20], where structural MRI is used for an initial registration and then diffusion tensor information is mapped based on the resulting transformation. This is mainly performed to reduce shape variance and to maintain direction consistency.

Consequently, fabric tensor mapping is modeled as the coordinate transform $T:R^{3X3}\to R^{3X3}$ involved in transforming the fabric tensor ***M*** from the space of the femur atlas image to the space of the patient’s femur image. The image registration process involves global and local deformations, which can be decomposed into stretch and rotation components. Understanding the impact of different components of deformations on tensor mapping becomes essential. In this regard, we have chosen five different tensor mapping schemes reflecting different degrees of freedom of the transformation used for fabric tensor mapping. For concision, they are henceforth referred to as:

1. *No Rotation (NR)*: Fabric tensor mapping involves only translation, which is a direct mapping from the femur atlas to the patient’s image. After image registration, point correspondences are established between patient femur and femur atlas. If ***M*** represents the computed fabric tensor from the femur atlas HRpQCT image, then tensor mapping based on **N**o **R**otation, termed here *NR*, is
  $$\begin{array}{c} M^{\prime }=IMI^{T}=M. \end{array}$$ (17)
  We note that inclusion of this mapping method is meant to show the impact of tensor mapping and its advantages.
2. *Affine Rotation (AR)*: Fabric tensor mapping involves affine rotation, which is a global transformation. After image registration between the patient image and the atlas image, the affine rotation matrix ***R***_***A***_ is derived from the deformation gradient ***F***. If ***M*** represents the computed fabric tensor from the femur atlas HRpQCT image, then tensor mapping based on **A**ffine **R**otation, termed here *AR*, is
  $$\begin{array}{c} M^{\prime }=R_{A}MR_{A}^{T}. \end{array}$$ (18)
  We note that tensor mapping by *AR* will not alter the eigen-values *m*_*i*_ but only eigen-vector ***m***_***i***_ of ***M***.
3. *Affine Deformation (AD)*: Fabric tensor mapping involves affine deformation, which is a combination of an affine rotation matrix and an affine stretch tensor, and it is a global transformation. After image registration between the patient image and the atlas image, the affine rotation matrix ***R***_***A***_ and affine stretch tensor ***V***_***A***_ is derived from the deformation gradient ***F***. Then, the affine deformation gradient ***F***_***A***_ = ***V***_***A***_***R***_***A***_ is computed. If ***M*** represents the computed tensor from the femur atlas HRpQCT image, then tensor mapping based on **A**ffine **D**eformation, termed here *AD*, is
  $$\begin{array}{c} M^{\prime }=F_{A}MF_{A}^{-1}. \end{array}$$ (19)
4. *Complete Rotation (CR)*: Fabric tensor mapping involves complete rotation, which is a combination of an affine rotation matrix and a B-spline rotation matrix. This is a local transformation. After image registration between the patient image and the atlas image, the affine rotation matrix ***R***_***A***_ and B-spline rotation matrix ^*j*^***R***_***B***_ is derived from the deformation gradient ^*j*^***F***. Then, the complete rotation matrix ^*j*^***R*** = ^*j*^***R***_***B***_ ∘ ***R***_***A***_ is computed. If ^*j*^***M*** represents the computed tensor from the femur atlas HRpQCT image, then tensor mapping based on **C**omplete **R**otation, termed here *CR*, is
  $$\begin{array}{c} {}^{j}M^{\prime }={}^{j}R{}^{j}M{}^{j}R^{T}. \end{array}$$ (20)
  We note that tensor mapping by *CR* will not alter the eigen-values *m*_*i*_ but only eigen-vector ***m***_***i***_ of ***M***.
5. *Complete Deformation (CD)*: Fabric tensor mapping involves complete deformation, which is a combination of the complete rotation matrix and the complete stretch tensor. It is also a local transformation. After image registration between the patient image and the atlas image, the deformation gradient or complete deformation gradient ^*j*^***F*** is computed. If ^*j*^***M*** represents the computed tensor from the femur atlas HRpQCT image, then tensor mapping based on **C**omplete **D**eformation, termed here *CD*, is
  $$\begin{array}{c} {}^{j}M^{\prime }={}^{j}F{}^{j}M{}^{j}F^{-1}. \end{array}$$ (21)

#### Fabric atlas

**Fig 3:**
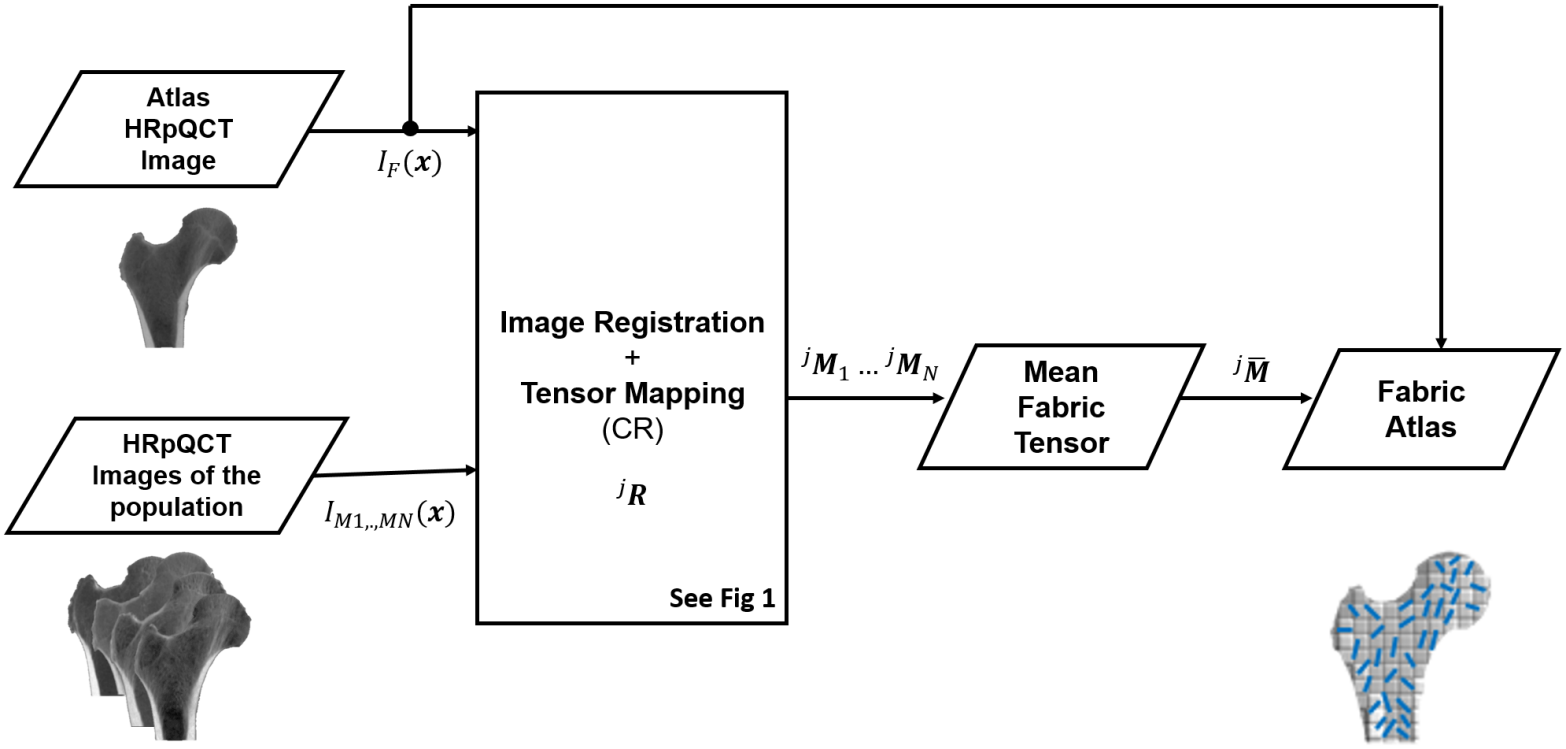
Overview of the construction of fabric atlas (femur atlas model with mean fabric) is presented. A HRpQCT femur image of the population is registered to the femur atlas HRpQCT image and point correspondence is established. Based on the inverse transform, fabric tensor is computed using MIL method. The computed fabric tensor is then mapped to the atlas femur HRpQCT image by CR tensor mapping method. The process is repeated for all the femurs of the population. Fabric atlas is constructed by averaging all the mapped fabric from each femur of the population.

In this section we present the methodology employed to construct a population-based atlas of fabric information. Differently from the diverse femur atlases described in section, a fabric atlas refers to a femur atlas model with a mean fabric tensor distribution. The overview of the construction of fabric atlas is presented in Fig 3. We follow a similar strategy as in cardiac DTI imaging for statistical analysis of cardiac fibres [21, 22]. Initially, a femur atlas HRpQCT image is chosen from the population. Image registration is performed between the femur atlas HRpQCT image *I*_*F*_ and another femur HRpQCT image of the population *I*_*M*_. For each control point *j* of the femur atlas, the corresponding control point *I*_*M*_(***T***^−1^(^*j*^ ***x***)) is found, and fabric tensor ^***j***^***M*** is computed following the procedure described in previous section. The computed fabric tensors from all control points are then mapped to the femur atlas HRpQCT image. Mapping is performed by CR tensor mapping method, as it yielded best results compared to other tensor mapping methods (see result section). The same procedure is repeated for the rest of the femur HRpQCT images *I*_*M*1_, *I*_*M*2_, …*I*_*MN*_ of the population and the respective fabric tensors ^***j***^***M***_**1**_, ^***j***^***M***_**2**_, ….^***j***^***M***_***N***_ are computed. Then, the mean fabric tensor at each control point *j* is computed by averaging

$$\begin{array}{c} \bar{{}^{j}M}=\frac{1}{N}\sum_{n=1}^{N}\left({}^{j}R{}^{j}M{}^{j}R^{T}\right). \end{array}$$ (22)

The resulting mean fabric tensor is an arithmetic synthetic fabric tensor distribution that is mapped on the femur atlas HRpQCT image (MTP) being closest to the synthetic average femur, as described in previous section. Along with the other femur atlases presented in previous section, the resulting fabric atlas will be evaluated for prediction of patient femur fabric information, using the evaluation metrics presented in the next section.

## Materials and experiments

### Datasource

The study was performed on a database of pairs of QCT and HRpQCT images of human proximal femora. The database comprises 36 pairs (17 males, 19 females with age 76±12 years, range 46–96 years) and were obtained from a previous study [4]. In summary, each femur was scanned with a calibration phantom (BDC Phantom, QMR Gmbh, Germany) in a clinical QCT (Brillance64, Phillips, Germany, intensity: 100 *mA*, voltage: 120 *kV*, voxel size: 0.33 × 0.33 × 1.00 *mm*^3^), and HRpQCT (Xtreme CT, Scanco, Switzerland, intensity: 900 *μA*, voltage: 60 *kVp*, voxel size: 0.082 × 0.082 × 0.082 *mm*^3^). The QCT images were rescaled to an isotropic voxel spacing (0.33 × 0.33 × 0.33 *mm*^3^) and were rigidly registered to the corresponding HRpQCT images. From the HRpQCT images, the cortical bone was masked out according to the procedure reported in [23].

#### Femur morphology

**Fig 4:**
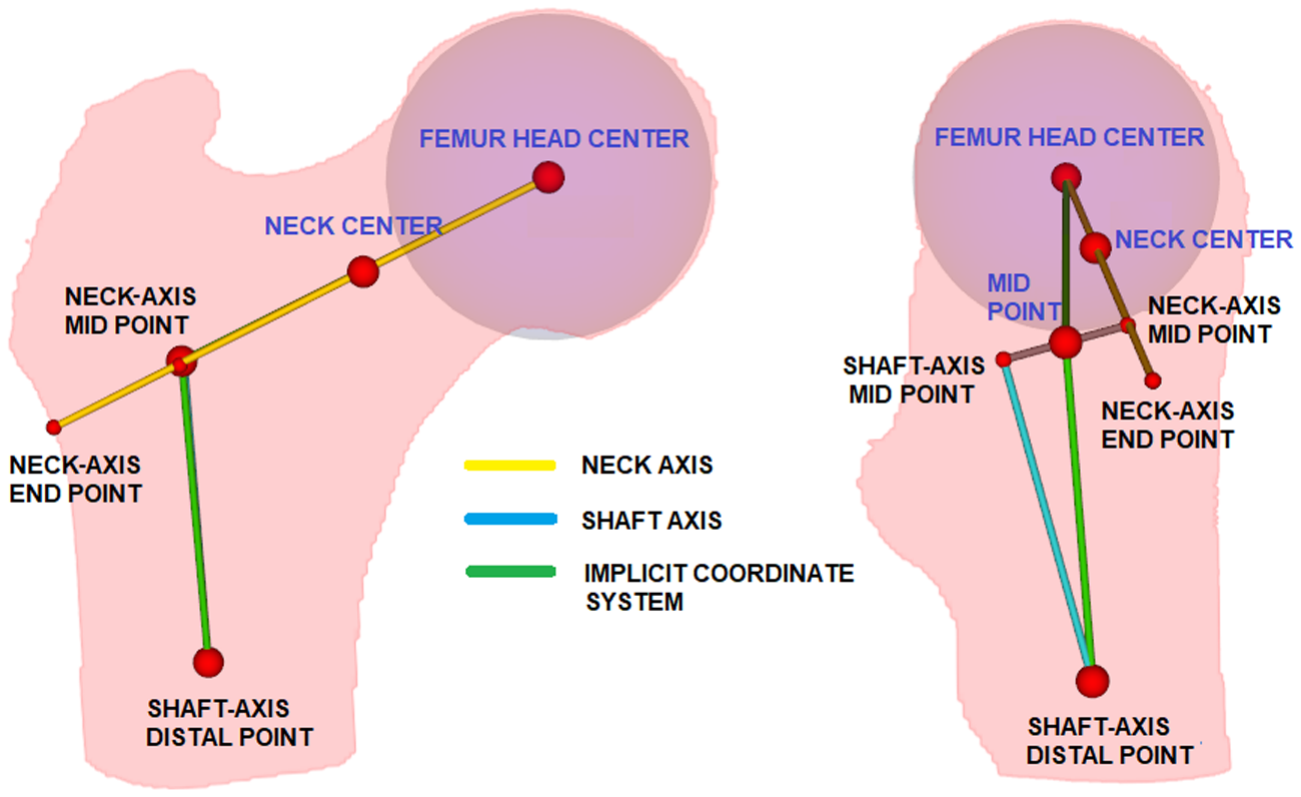
Implicit coordinate system of the human femur. Left: Coronal view. Right: Sagittal view. The red dots indicates the key points found on the femur. The green line indicates the constructed implicit coordinate system. Labels in blue color are used for morphology study.

In order to assess how representative the selected database is with respect to the shape variability of the femur anatomy, a femur morphology study was first performed. To this end, an implicit coordinate system of the femur was constructed as shown in Fig 4. First, the femoral head center is defined by a mass center of a spherical region with maximal cross-section area. The neck axis is then computed by following the procedure reported by Kang et al. [24, 25]. In short, the radius of the spherical region of the femoral head is enlarged by 25%, and an initial neck center is defined. Using Powell’s optimization [26], the femoral neck center is computed, and the neck axis is defined as the line between femoral head center and femoral neck center (see Fig 4). The intersection point between the neck axis and the lateral surface of the femur is defined as the neck-axis-end-point. Then, the mass center of slices distal to this point are computed, followed by RANSAC fitting [27] to define the shaft axis. Generally, as the neck and shaft axes do not intersect, a mid point is defined as the shortest distance between the neck and shaft axes. The most distal point of the shaft axis is chosen as shaft-axis-distal-point. An implicit coordinate system is constructed by connecting femoral head center, mid point and shaft-axis-distal-point. As morphological parameters we calculated known shape descriptors of the femur, such as the caput-collum-diaphyseal angle (CCD), femoral head diameter, and distances describing the femoral neck anatomy. Femur morphology was computed for the total and sex-specific populations, and are summarized in Table 1. Between the Sex-specific populations, the morphology of the femurs were not found to be statistically significant(p>0.05).

**Table 1:** Morphology of the femur population.

Population	Total	Male	Female
CCD(°)	128±7	129.5±8	126.5±6
Distance Femoral Head Center—Neck Center(mm)	26.7±3.8	27.6±3.8	26±3.7
Distance Femoral Head Center—Mid Point(mm)	47.7±5.9	47.6±6.4	47.9±5.4
Femoral Head Diameter(mm)	46.4±4.0	47.4±3.5	45.5±4.2

#### Image pre-processing

Image pre-processing was performed on femur images to correct its shaft length, as the acquired images have varying shaft length. This step was also performed to ensure that the image registration step is not affected by differences in the anatomy. The shaft region of the femur was chopped such that the ratio between the distance femoral head center and mid point, and mid point and shaft-axis-distal-point (see Fig 4) equals 0.7, which was found empirically in order to yield an stable image registration. All the femurs were rigidly aligned with mid point as center.

### Experimental design

We designed two experiments to answer the three open questions in registration-based bone fabric prediction, summarized below:

1. Impact of femur atlas selection and sex considerations.
2. Impact of the fabric mapping transformation on fabric prediction accuracy
3. Potential of population-wide and sex-specific mean fabric atlases

In the first experiment, we combined in the evaluation the analysis of using different femur atlases (section) as well as different fabric tensor mapping transformations (section). In the second experiment, we evaluated the accuracy of predicting femur fabric by means of a femur atlas featuring a synthetically generated mean fabric (section) or its corresponding fabric tensor, as extracted from its HRpQCT image pair.

#### Evaluation scheme and metrics

For numerical evaluation a leave-one-out (LOO) strategy was followed. Specifically, a femur is chosen from the population as the patient’s femur and its counterpart (left or right) is removed from the population to remove bias in the analysis. At each control point *j*, the predicted fabric tensor for the patient’s femur QCT image is represented as $\hat{M}$ with eigenvalues $\hat{m_{3}}$, $\hat{m_{2}}$, $\hat{m_{1}}$ and eigenvectors $\hat{m_{3}}$, $\hat{m_{2}}$, $\hat{m_{1}}$ and the corresponding ground truth fabric tensor is computed from the patient’s femur HRpQCT image and is represented as ***M*** with eigenvalues *m*_3_, *m*_2_, *m*_1_ and eigenvectors ***m***_**3**_, ***m***_**2**_, ***m***_**1**_. The process is repeated for each image in the database (N = 72 images), for each femur atlas (N = 6, section), and for each fabric mapping transformation (N = 5, section), leading to 5′040 (72 × 70) image registrations.

To evaluate the accuracy of the predicted femur fabric, we adopted the same evaluation metric as described in [6]. Namely, tensor norm error (*TN*_*error*_), degree of anisotropy error (*DA*_*error*_) and angular error of the principal tensor direction (*PTD*_*error*_), are computed as follows:

$$\begin{array}{c} TN_{error}=\frac{∥\hat{M}-M∥}{∥M∥},\;DA_{error}=\frac{|\hat{DA}-DA|}{DA}\;\text{and}\;PTD_{error}=\arccos\left(\hat{m_{3}},m_{3}\right), \end{array}$$ (23)

where the predicted, and ground-truth degree of anisotropy (*DA*) and ($\hat{DA}$), respectively, are computed as

$$\begin{array}{c} \hat{DA}=\frac{\hat{m_{3}}}{\hat{m_{1}}}\;\text{and}\;DA=\frac{m_{3}}{m_{1}}. \end{array}$$ (24)

The average error for each evaluation metric was computed for all the control points *J* and for all images in the LOO study, and were used as the base for comparison.

### Results

#### Impact of femur atlas selection and sex considerations

**Fig 5:**
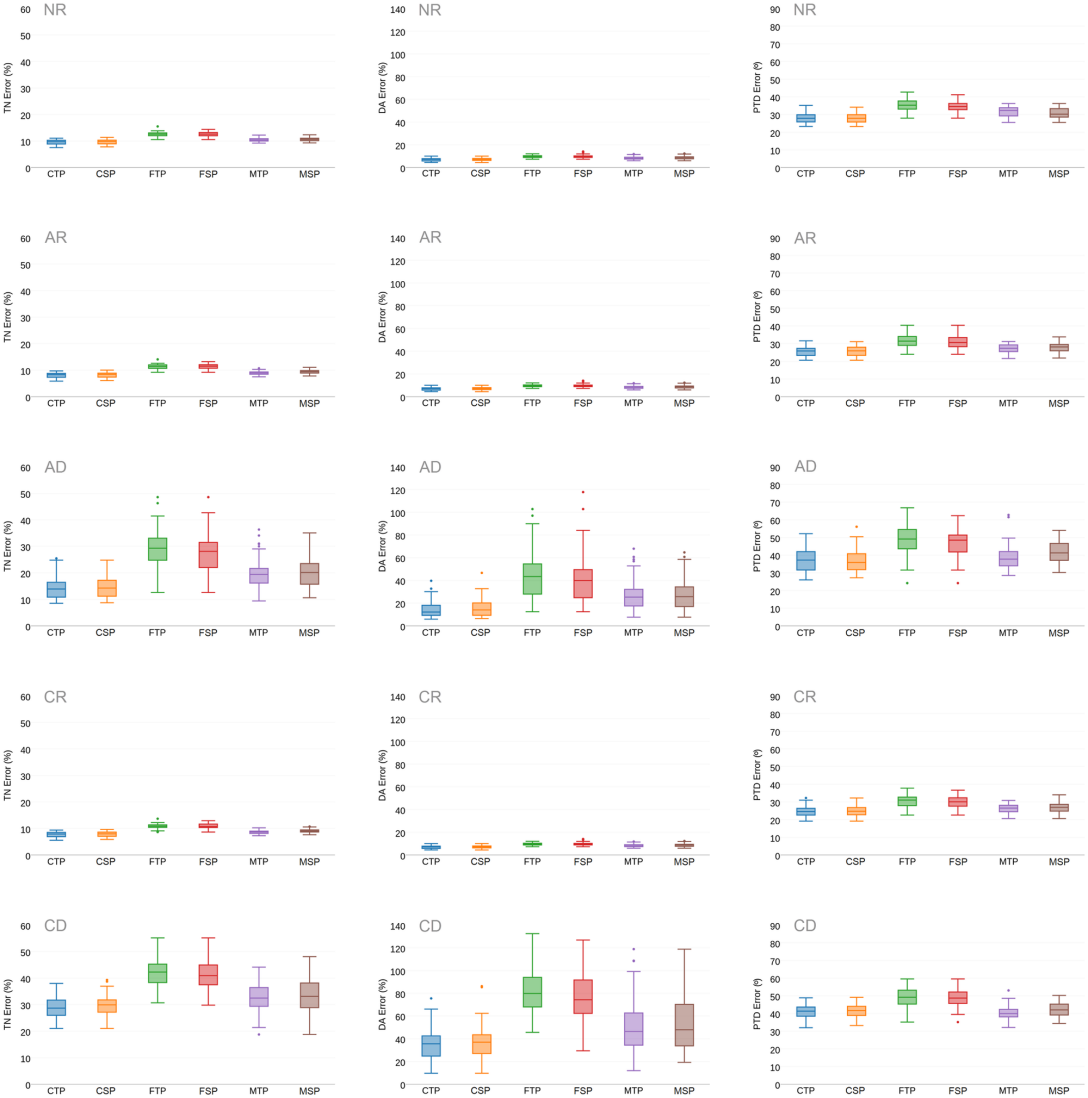
Summary of prediction error for different sets of combination of tensor mapping methods and femur atlases. Rows represents different tensor mapping methods. First Row: Tensor mapping by NR, Second Row: Tensor mapping by AR, Third Row: Tensor mapping by AD, Fourth Row: Tensor mapping by CR, Fifth Row: Tensor mapping by CD for different femur atlases. Columns represents the error metrics. First Column: TN Error, Second Column: DA error, Third Column: PTD error. Acronyms: CTP—Closest to patient femur in the Total Population, CSP—Closest to patient femur in the Sex-specific Population, FTP—Farthest to patient femur in the Total Population, FSP—Farthest to patient femur in the Sex-specific Population, MTP—Mean femur of the Total Population, MSP—Mean femur of the Sex-specific Population, NR—No rotation, AR—Affine Rotation, AD—Affine Deformation, CR—Complete Rotation, CD—Complete Deformation.

We first present in Fig 5 overall results for all three evaluation metrics, for each femur atlas and fabric tensor mapping transformation. Regarding the selection of the femur atlas, as expected the farthest femur atlases (*FTP and FSP*) yielded the highest errors, followed by the mean femur atlases (*MTP and MSP*). We remark that the selection of FTP and FSP was motivated to reflect a potential worst-case scenario and to test the hypothesis that an atlas should be as similar as possible to the patient image on which fabric is predicted. Results on all metrics showed that choosing the closest femur atlases (*CTP*, *CSP*) yields the lowest errors, which verifies the importance of the femur atlas selection.

Regarding sex, no statistically significant differences for all three metrics were found (p>0.05) between choosing atlases from the total population (*CTP*, *FTP*, *MTP*) or sex-specific ones (*CSP*, *FSP*, *MSP*). This results suggests that it might not be necessary to create sex-specific femur atlases when predicting femur fabric.

#### Impact of the fabric mapping transformation on fabric prediction accuracy

**Fig 6:**
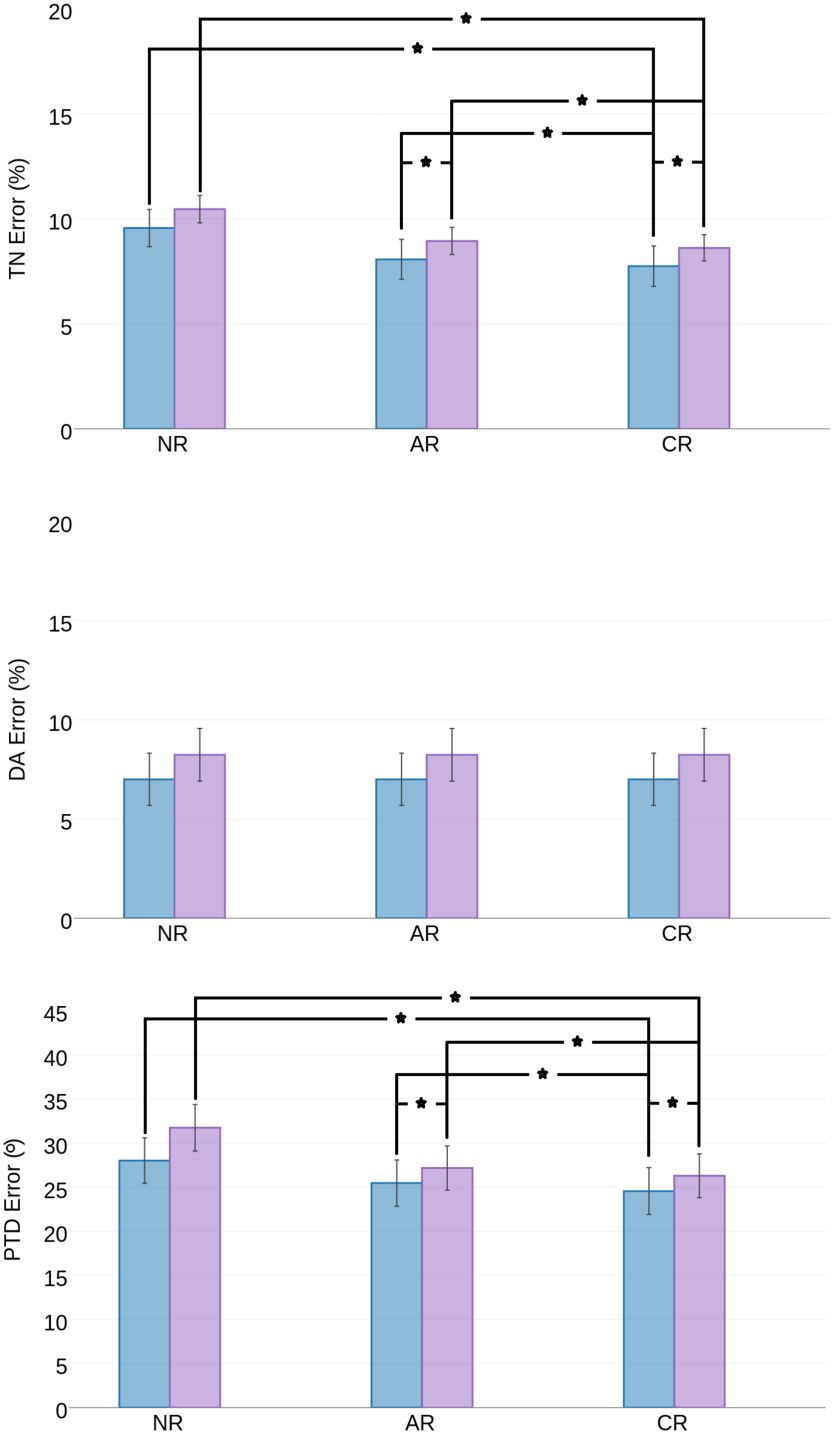
Summary of prediction error for different sets of combination of fabric tensor mapping methods involving only rotation component (NR, AR, CR) and femur atlases(CTP in blue, MTP in pink). First row: TN Error, Second row: DA Error, Third row: PTD Error. The DA Error remains the same, because the tensor mapping methods involves only rotation component and hence doesn’t alter the eigen values. Acronyms: CTP—Closest to patient femur in the Total Population, MTP—Mean femur of the Total Population, NR—No rotation, AR—Affine Rotation, CR—Complete Rotation (*—p < 0.05).

Regarding the impact of the fabric mapping transformation, results presented in Fig 5 show that fabric tensor mapping methods involving only rotation components (*CR*,*AR*) produce lower errors than tensor mapping methods involving both rotation and stretch components (*CD*,*AD*). Among the methods relying only on rotation, fabric tensor mapping by *CR* yielded the lowest error, followed by *AR* and *NR*. However, only the *TN*_*error*_ and *PTD*_*error*_ were found to be significantly different, as shown in Fig 6. This is due to the fact that fabric tensor mapping methods involving only a rotation component do not alter the eigenvalues, and hence *DA*_*error*_ remained the same.

Relative to the selected femur atlas, using *CR* fabric tensor mapping in combination with *CTP* yielded *TN*_*error*_ = 7.3±0.9%, *DA*_*error*_ = 6.6±1.3%, and *PTD*_*error*_ = 25±2°). These results compare favorably to those yielded when using *MTP* as femur atlas, with *TN*_*error*_ = 7.7±1.0%, *DA*_*error*_ = 7.0±1.4%, and *PTD*_*error*_ = 25±2°. Nonetheless, it is remarked that while CTP requires image registration for each image of the database to calculate the distance metric *DM*, *MTP* is computed only once and does not require further computations across the database. Fig 6 focuses on analyzing the performance for these two femur atlases (*CTP*,*MTP*), and fabric tensor mapping methods based on a rotation component (*NR*, *AR*, *CR*). Statistically significant differences (p>0.05) were found between NR and CR, and between CR and AR, but not between NR and AR, confirming the value of using CR as preferred fabric tensor mapping transformation.

#### Potential of population-wide and sex-specific mean fabric atlases

**Fig 7:**
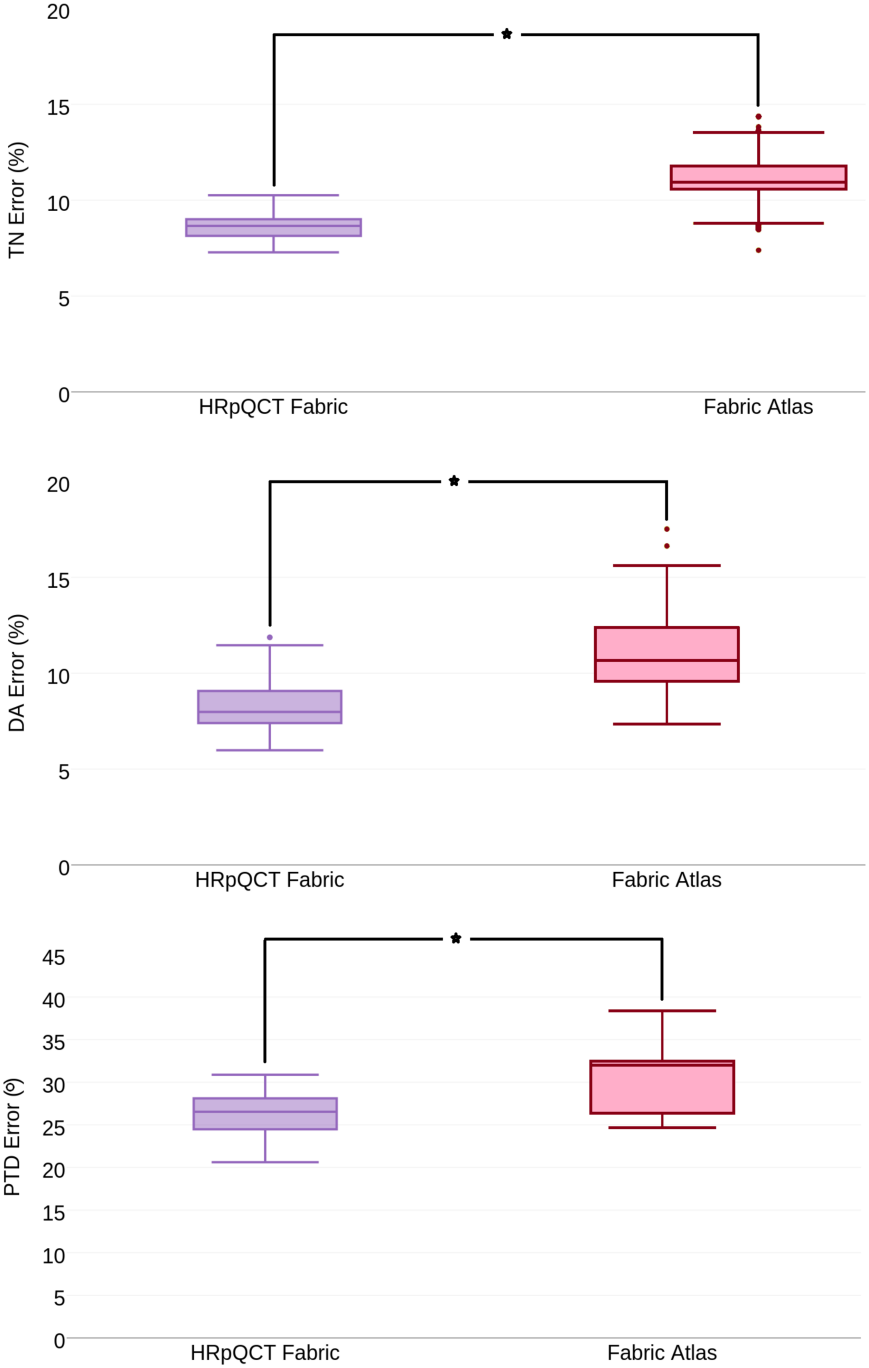
Summary of prediction error, comparing bone fabric prediction by femur atlas with HRpQCT fabric and fabric atlas. MTP was chosen femur atlas. The mapping of bone fabric was performed by CR tensor mapping method. First row: TN Error, Second row: DA Error, Third row: PTD Error. Acronyms: MTP—Mean femur of the Total Population, CR—Complete Rotation (*—p < 0.05).

Fig 7, shows fabric prediction errors for all three evaluation metrics, when predicting femur fabric by means of a femur atlas featuring a synthetically generated mean fabric or by its corresponding real fabric tensor, as extracted from its HRpQCT image pair. In this experiment, *MTP* and *CR* were chosen as femur atlas and fabric tensor mapping method, respectively. We found that using the synthetically generated fabric atlas yielded higher error than using the real fabric from the corresponding HRpQCT fabric. A statistical difference was found (p < 0.05).

#### Spatial and bone mineral density based evaluation of femur fabric prediction

**Fig 8:**
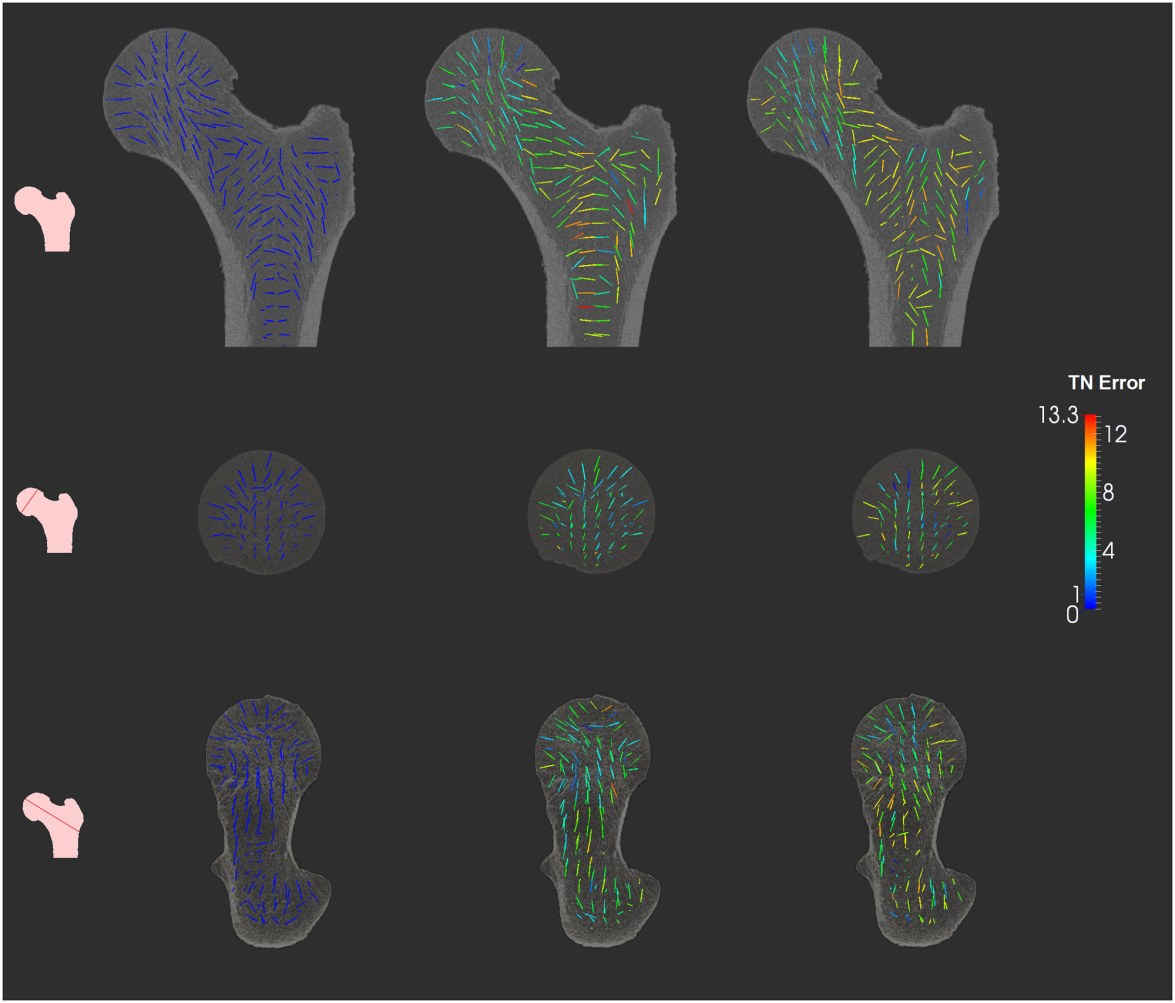
Illustration of bone fabric prediction accuracy achieved on a test case femur. Left column: the lines indicate the principal orientation of the tensors computed from the test case femur’s HRpQCT image. Middle column: the lines indicate the principal orientation of the tensors mapped from the CTP femur atlas and CR tensor mapping method. Right column: the lines indicate the principal orientation of the tensors mapped from the MTP femur atlas and CR tensor mapping method. The colors correspond to the TN error. Rows shows different planes and the small femur image with red line shows the plane being visualized. Acronyms: CTP—Closest to patient femur in the Total Population, MTP—Mean femur of the Total Population, CR—Complete Rotation.

We performed a spatial analysis of fabric prediction performance to analyze how the prediction errors are spatially distributed. Fig 8, shows in three different planes, the prediction of bone fabric for an example case using selected femur atlases *CTP and MTP*, and *CR* as fabric tensor mapping method. It is observed that the *TN*_*error*_ varies widely across different regions of the femur. We observed that lower error are observed across the main loading direction and in femoral head regions. Higher errors were observed in the shaft and in lower trochanter regions.

**Fig 9:**
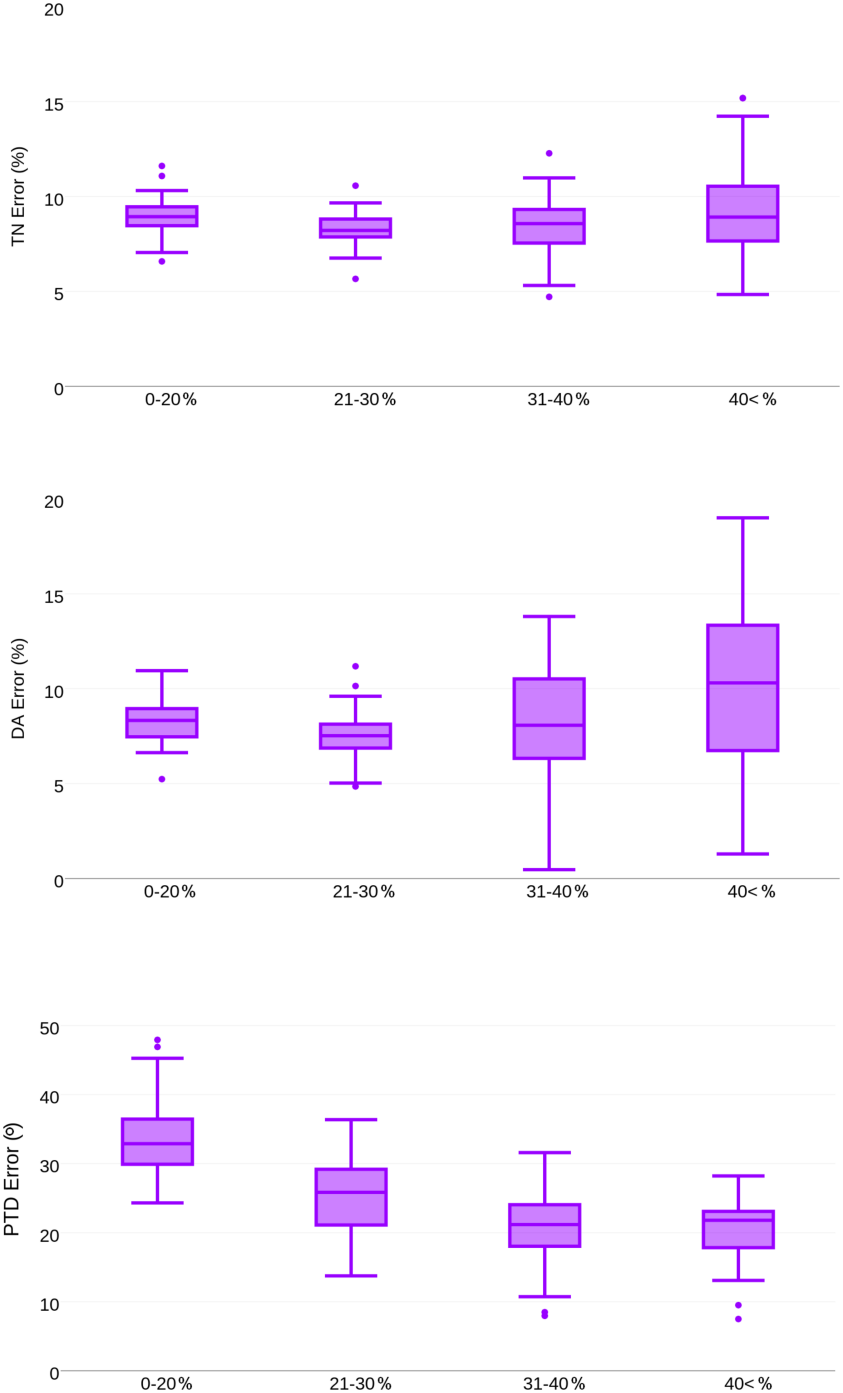
Summary of prediction error comparing bone fabric prediction for different BVTV bins. MTP was chosen as the femur atlas. The mapping of bone fabric was performed by CR tensor mapping method. First row: TN Error, Second row: DA Error, Third row: PTD Error. Acronyms: MTP—Mean femur of the Total Population, CR—Complete Rotation.

Finally, as the registration process is driven by image intensity information we were interested to analyze whether there is a correlation between bone mineral density and fabric prediction error. Fig 9 shows for each metric bone fabric prediction errors for different Bone Volume over Total Volume (BVTV) bins. In this experiment, *MTP* and *CR* were chosen as femur atlas and fabric tensor mapping method, respectively. We observed increasing errors for *TN*_*error*_ and *DA*_*error*_ in regions of moderate to high BVTV, whereas lower *PTD*_*error*_ errors were found for moderate to high BVTV regions.

## Discussion

In this study we propose a novel registration-based estimation of bone fabric directly from clinical QCT images. It is designed to preserve tensor properties of bone fabric and to map bone fabric by a global and local decomposition of the gradient of a non-rigid image registration transformation. We analyzed the importance of the fabric tensor mapping transformation as well as the femur atlas used to map the fabric information into a target QCT image. We further evaluated and reported the performance of a population-, and sex-specific atlas of bone fabric used within the proposed registration-based fabric estimation approach.

The entire study was performed on a database of 36 pairs of human proximal femora [4], for which the results of the morphology analysis suggest that the femurs used in the present study are representative of femurs from other studies [28].

### Importance of femur atlas selection

Regarding different femur atlases, it becomes clear from Fig 5 that the farthest to the patient’s femur, FTP and FSP, yielded rather poor results. Conversely, the closest to the patient’s femur, CTP and CSP, yielded the best results, which allow us to conclude that bone fabric prediction based on image registration is sensitive to the selected femur atlas. These results are in agreement with the strategy presented in [3] where a femur database and selection scheme was originally presented. From a physiological loading point of view, it is indeed expected that differences in bone anatomy have an effect on the underlying bone fabric [29]. As reported in Table 1 as well as in previous studies regarding bone femur morphology [28], such difference in bone anatomy is observed through parameters such as the CCD angle and neck length. However, further FE simulations on a representative population are required to assess the impact of femur atlas selection on bone strength prediction.

In addition, regarding sex considerations, results suggests that there is no major benefit in employing sex-specific femur atlases for fabric prediction. The probable reason for this finding is that the variability in femoral shape (in terms of DM) between sex-specific populations is smaller than 3% of the population shape variability. Table 1 supports this statement where the femurs’ morphological variables of females and males are in average similar (p>0.05).

Interestingly, results presented in Figs 5 and 6 suggest while the highest fabric prediction accuracy is attained with CTP, followed by MTP, their differences in accuracy are often statistically significant, but quantitatively the results are rather close. In this regard, one important practical limitation of using CTP involves computing the closest femur image (in terms of DM) in the population. In practice, such computations are prohibitive for large databases. On the contrary, MTP is computed once and if needed, it can be updated for an extended or different population database.

### Impact of fabric tensor mapping

Looking at different fabric tensor mapping methods, results indicate that tensor mapping by AR and CR performs better than CD and AD. The DA error clearly supports the conclusion that the stretch component of CD and AD tends to alter the bone fabric excessively. The importance of the tensor mapping method becomes also clear from Fig 6, where the tensor mapping by NR does not improve fabric prediction in terms of TN and PTD (p<0.05). Conversely, fabric tensor mapping by AR does improve fabric prediction (p<0.05), and fabric tensor mapping by CR yields the best fabric prediction accuracy (p<0.05).

On the other hand, as rotation-only mapping approaches do not alter the eigenvalues of fabric tensors, these approaches are not capable of predicting DA. These results suggest that the degrees of freedom of the chosen transformation model plays an important role, and a trade-off between accuracy of predicting fabric orientation and DA needs to be considered when using rotation-only mapping schemes.

Turning to the concept of employing a synthetically generated fabric atlas, results presented in Fig 7 suggests that bone fabric predictions are considerably less accurate than when using the real fabric of the HRpQCT image. One possible reason of this is the fact that the femur atlas stems from a real bone image, which is then combined with a computed (i.e synthetic) mean fabric distribution. Such combination might not fully characterize the interplay between bone morphology and fabric distribution, as naturally occurs for a real bone image, where bone fabric and bone morphology are interrelated [29]. Although algorithms exist to compute a mean femur atlas [30], our experiments yielded an over-smoothed synthetic image not preserving the required image quality inherent of HRpQCT. Further research on atlas construction approaches specifically designed to deal with tensorial information, such as [21, 22], might provide improvements to the creation of high resolution femur atlases.

### Spatial and BVTV based analysis of fabric prediction accuracy

The spatial distribution of the tensor norm error in Fig 8 shows that the bone fabric prediction accuracy varies widely across regions. In particular, the femoral head and the main loading trajectory (which is of primary interest for FE analysis) present higher fabric prediction accuracy than the inter-trochanteric region. This finding may be due to the inability of the image registration approach to handle properly regions of lower BV/TV. From a physiological point of view, the higher BV/TV relates to bone micro-architecture oriented along the principal stresses acting on the femur and forming characteristic trajectories [31]. To further clarify this issue, the bone fabric prediction was analyzed with respect to BV/TV. Fig 9 indicates indeed better predictions of the major fabric orientation in regions of high BV/TV.

### Limitations of the present study

Some limitations of this study have to be mentioned. First, we use a distance metric (DM) for selection of the femur atlas that does not not consider any anthropometric parameters or ethnic variation. Their inclusion in the analysis would be of great interest for patient-specific FE analysis. However, such information was not available for the database used in this study.

Second, the processing time of the present approach for one femur takes 40 min. The time was measured on a desktop with the application running single-threaded on a 3.20 GHz Intel Core i7 processor. Such computation time is relatively high compared to other machine learning based approaches for bone fabric predictions [6, 7]. However, this is a major common disadvantage of all image-based registration approaches.

Third, the optimal mapping approach, CR, is not capable of improving the prediction of DA. One of the possible way to address this problem would be the use of poly-affine registration [32, 33], where a set of affine transformations is employed to characterize spatial transformations with a low number of parameters. As described, our experiments suggest that a trade-off between flexibility of the transformation model to morph the atlas image onto the patient image, and preservation of bone fabric information exists. Similarly, the use of dedicated registration algorithms encoding specific properties linked to the anatomy or disease in study (e.g. [34]), or registration approaches previously proposed for Diffusion Tensor Imaging (DTI) (e.g. [21, 22]) might provide a better fabric prediction based on image-registration approaches.

### Comparison to previous approaches

Although previous approaches have used different datasets and sample sizes (Taghizadeh et.al [2] N = 10, Chandran et.al [7] N = 30, Lekadir et.al [6] N = 33), some quantitative comparisons are worth mentioning. The present study yielded lower TN and PTD errors than the one of [2], where a TN error of 14.8 ± 1.5% and a PTD error of 29.7 ± 3.3° were reported. On the other hand, studies based on machine learning approaches, such as [7] reported a TN error of 6 ± 2%, and a PTD error of 19 ± 7°, and [6] reported a TN error of 7 ± 1% and a PTD error of 15.6 ± 2.3°, which are comparable for TN but lower for PTD compared to the registration-based method explored in the present study. Regarding prediction of DA, the studies of [7] and [6] reported DA error (6 ± 2% and of 7 ± 1% respectively), which are comparable to the ones obtained with registration-based methods.

The present study lacks the experimental data to validate the role of predicted bone fabric in computational models for calculation of bone strength. However, previously reported bone fabric prediction accuracies [3, 6, 29, 35] are in the similar range of prediction accuracy reported here. Hence, we expect corresponding improvements in bone strength predictions.

## Conclusion

In conclusion, we proposed a novel image-registration based femur fabric prediction directly from clinical QCT image. The methodology is robust and favorably compares to previous state of the art registration-based method for femur fabric prediction. Furthermore, we present a comprehensive analysis of key components of the registration-based approach for bone fabric prediction in the proximal femur. From the results, we could answer three open questions. First, compromising between accuracy and computing time, the optimal femur atlas corresponds to the mean of the total population (MTP). Second, the best tensor mapping method is provided by complete rotation (CR). Third, a population average fabric atlas produced higher errors in fabric prediction than employing directly MTP and CR, and hence it is not recommended. By employing MTP, registration with a whole database of femurs becomes unnecessary and reduces considerably computational time.

The reported findings are promising for a clinical implementation and exploitation for patient-specific analysis as it is has potential to leverage bone architectural information directly from standard clinical imaging. Moreover, while image registration algorithms are improving we note on the importance of designing clinically- and task-oriented image registration pipelines. In this sense, the set of recommendations generated from this study are expected to guide the development of dedicated image based assessment methodologies of bone architecture from clinical imaging. The impact of the identified image-registration methodology on the prediction of hip strength by finite element analysis will be evaluated in future work.
